# Silenced Survivors: A Systematic Review of the Barriers to Reporting, Investigating, Prosecuting, and Sentencing of Adult Female Rape and Sexual Assault

**DOI:** 10.1177/15248380241261404

**Published:** 2024-07-30

**Authors:** Michelle Wieberneit, Sascha Thal, Joseph Clare, Lies Notebaert, Hilde Tubex

**Affiliations:** 1 The University of Western Australia, Australia; 2 Curtin University, Australia

**Keywords:** adult women, sexual assault, rape, criminal justice system, barriers

## Abstract

Sexual victimization of adult women remains an underreported crime. This systematic review identified and synthesized the barriers to reporting, investigating, prosecuting, and sentencing cases of sexual assault and rape against adult women in Western countries. Following the Preferred Reporting Items for Systematic Reviews and Meta-Analyses guidelines, a comprehensive search was conducted on August 3, 2023, across databases including PsycINFO, MEDLINE, Cochrane Library, Scopus, ProQuest Central, Web of Science, MedNar, and ProQuest Dissertations & Theses. Studies meeting the inclusion criteria provided relevant information on the decision not to formally disclose, investigate, prosecute, or convict incidents of sexual assault and rape of adult women. We included 28 studies and identified 70 barriers in total. Identified barriers were most prevalent to reporting, followed by investigating, prosecuting, and, lastly, sentencing. Key themes in the barriers included lack of trust in the criminal justice system, internal reactions, rape myths and societal norms, and perpetrator characteristics. The identified barriers emphasize an urgent need for reform of the criminal justice system’s response to sexual assault and rape. Prioritizing victim-survivors’ needs, enhancing transparency of the criminal justice system, and addressing attrition rates are crucial. Future studies need to engage with diverse population to address all victim-survivors’ needs and provide further insights into the challenges across all stages of the criminal justice system to enhance the outcome of rape and sexual assault cases.

Sexual victimization among women continues to be a prominent and widespread concern worldwide, affecting nearly one-third of women who have encountered physical and/or sexual violence from an intimate partner, non-partner sexual violence, or both, at least once in their lifetime (World Health Organization, 2021). Sexual assault is commonly defined as engaging in physical sexual contact with a person without their consent (Aebi et al., 2010; Fileborn, 2011).

Rape and sexual assault are recognized as highly underreported crimes to law enforcement (Conroy & Cotter, 2017). Estimated reporting rates to the criminal justice system vary globally. For example, in the United States, approximately 31% of rape and sexual assault cases are reported (Morgan & Thompson, 2021), while in Australia, only 13% of incidents are formally reported to law enforcement (Australian Bureau of Statistics, 2021). As is discussed in the following sections with a focus on Western countries, the low rates of reporting can be attributed to barriers that women face in disclosing their sexual victimization to the criminal justice system, as well as challenges within the system that hinder the progression of cases and the attainment of convictions once a formal report has been filed.

## Barriers to Disclosure of Sexual Assault and Rape

Barriers to formally disclosing sexual victimization are multifaceted and involve psychological, sociocultural, and systemic factors. Orchowski et al. (2022) conducted a study analyzing tweets under the hashtag “WhyIDidntReport” during the highly publicized testimonies of Dr. Christine Blasey Ford and Supreme Court nominee Judge Brett Kavanaugh in the United States in September 2018 (Fortin, 2018). They categorized the identified barriers into individual, interpersonal, and sociocultural levels. Individual barriers included fear of disbelief, self-blame, shock, betrayal, and concerns about privacy. Interpersonal barriers encompassed the perpetrator relationship, negative reactions to disclosure, and power imbalances. Sociocultural barriers involved the belief that reporting would not lead to justice and societal norms impacting women’s reporting.

In a similar vein, Reich et al. (2022) also examined tweets under the hashtag “WhyIDidntReport” and identified seven themes related to stigmatizing and criminal factors. Stigmatizing barriers encompassed internal reactions such as shame, guilt, and fear, negative reactions from others, adherence to rape myths and societal norms, and protecting the perpetrator or others. Criminal factors included a perceived lack of evidence, such as memory gaps and delayed reporting, as well as power imbalances in perpetrator characteristics. These findings align with previous research, including the fear of consequences on relationships and blame (Logan et al., 2005).

### Barriers to the Investigation, Prosecution, and Sentencing of Sexual Assault and Rape

The few victim-survivors reporting their case to police often experience a “justice gap” (Temkin & Krahé, 2008), where the disparity between reported cases and cases that result in prosecution and conviction is significant (Bright et al., 2021; Rotenberg, 2017)—a process known as attrition. While attrition impacts all types of crimes, the prevalence in cases of sexual assault and rape is more pronounced and intricate. Historic emphasis on victim credibility induces skepticism toward any victim-survivor who does not fit the stereotypical victim (Randall, 2010). The gendered idea of women being liars and how they should behave during trial based on the ideal of a rational and irrational person add further complexity (Smith, 2018a, 2018c). Similarly, racialized stereotypes about victim-survivors induce less sympathy (Foley et al., 1995), and lead victim-survivors to be perceived as more likely to have consented (Katz et al., 2017), and hypersexual (French, 2013). Global rates indicate high attrition and low conviction rates for sexual offenses including the United States (Alderden & Ullman, 2012), the United Kingdom (Hohl & Stanko, 2015), Denmark (Hansen et al., 2015; The Swedish National Council for Crime Prevention (Brå), 2020), South Africa (Machisa et al., 2023), Germany (Jehle, 2012; The Swedish National Council for Crime Prevention (Brå), 2020), New Zealand (New Zealand Ministry of Justice, 2023), and Australia (Australian Bureau of Statistics, 2021; Bright et al., 2021). Daly and Bouhours (2010) reported an average overall conviction rate of 15% for sexual offenses between 1970 and 2005 across the United States, Canada, Scotland, England, and Wales. Similarly, a study by Aebi and Linde (2012) analyzed trends in convictions for six offenses—intentional homicide, robbery, theft, rape, assault, and drug offenses—for several European countries between 1990 and 2006. They found the highest rate of conviction for intentional homicide and lowest for rape.

Prior research sought to illuminate the factors affecting attrition. Attrition can happen at three stages once a formal complaint has been filed: police decision-making, prosecutorial decision-making, and court outcomes (Jehle, 2012). Legal and extra-legal factors can affect the decision-making across all stages of the criminal justice system. Legal factors focus on the legal aspects of the case, including culpability of the defendant, evidence, and seriousness of the offense, whereas extra-legal factors are aspects of a case that do not have legal recognition to influence the response to a case, such as characteristics of the defendant and victim including their background, social status, and credibility (Alderden & Ullman, 2012; Frohmann, 1997; Kapardis, 1984).

Extra-legal factors affecting police (Gekoski et al., 2024; O’Neal & Hayes, 2020) and prosecutorial (St. George & Spohn, 2018) decision-making in cases of sexual violence include rape myths, which are “descriptive or prescriptive beliefs about rape (i.e., about its causes, context, consequences, perpetrators, victims, and their interaction) that serve to deny, downplay or justify sexual violence that men commit against women” (Bohner, 1998, p. 12). Rape myths shift the blame from the perpetrator to the victim-survivor (Burt, 1980). Examples of such beliefs are that women commonly lie about rape (Lonsway & Fitzgerald, 1994) and that most rapes are committed by strangers in a public place involving the use of physical force or threats of physical harm (Estrich, 1987). Factors, such as the voluntary consumption of alcohol by the complainant prior to the rape, perceived intoxication or lack of physical resistance of the complainant during the rape, and a history of consensual sex with the perpetrator may amplify victim-blaming attitudes (e.g., Murphy-Oikonen, Chambers et al., 2022; Murphy-Oikonen, McQueen et al., 2022; Venema, 2016). Moreover, the ethnicity of the suspect, with white males being more likely to escape further investigation, and the credibility of the complainant have been found to influence police decision-making (Alderden & Ullman, 2012; Hohl & Stanko, 2015). Prosecutorial decision-making has been found to be influenced by the complainant’s willingness to cooperate (Kennedy, 2018; Spohn & Tellis, 2019), whether the case is likely to result in a conviction (O’Neal et al., 2015), the complainant’s perceived credibility (Beichner & Spohn, 2005; Campbell et al., 2012), the presence of injuries (Alderden & Ullman, 2012; Fansher & Welsh, 2023; O’Neal et al., 2015), and whether the complainant and perpetrator were acquainted (Beichner & Spohn, 2005; Pattavina et al., 2021).

If a survivor decides to report the crime and the case moves to trial, the court proceedings can be re-traumatizing for the survivor (McDonald, 2020) and conviction rates remain low (Cossins, 2020). In countries like the United States, the United Kingdom, and Australia, this can be attributed, in part, to the adversarial nature of the legal system, where survivors often face challenges to their testimony and character during trials to weaken their case (Smith, 2018b), but also to a broader context of institutional distrust. This distrust stems from systemic issues, such as high workloads and lack of resourcing for investigators and investigative processes (Harding et al., 2024; Stanko, 2022) and procedural complexities (Boyer et al., 2018) that create significant hurdles for victim-survivors seeking justice. Research on extra-legal factors influencing judges’ decision-making is dearth. However, there is ample research indicating that extra-legal variables including rape myths affect jurors and their decision-making (for a review, see Leverick, 2020). In recent years, efforts have been made to address these issues and improve outcomes for victim-survivors, particularly in the wake of the #MeToo movement. For example, states in Australia are reviewing their criminal justice system responses to sexual violence to ensure victim-survivors are supported and receive a justifiable outcome (Attorney-General’s Department, n.d.; Commissioner for Victims of Crime, 2023). However, barriers persist within society and the criminal justice system, impeding the formal reporting and case outcome of sexual victimization.

### The Present Study

To date, no systematic review has been conducted to examine and synthesize the factors that serve as barriers to formally report sexual violence and the barriers that might affect the investigation, prosecution, and conviction of formally reported cases. Focusing on studies conducted in Western countries, our review expands prior reviews which have typically explored a set of barriers to the criminal justice system’s response to sexual assault and rape (e.g., Nitschke et al., 2019; Parratt & Pina, 2016; Sleath & Bull, 2017) by synthesizing barriers at all stages of the criminal justice system. All these barriers might contribute to the high attrition and low conviction rates. With the evolving landscape of sexual violence research and increased awareness and reforms specifically since the #MeToo movement in 2017 (Levy & Mattsson, 2023; National Women’s Law Center, 2023), our review focuses on research published between January 1, 2013, and August 3, 2023.

Our systematic review focuses specifically on sexual victimization, including threatened, attempted, or completed rape, as well as sexual assault. We defined rape as vaginal, oral, or anal penetration and sexual assault encompassing unwanted sexual contact with or without the use of force. It is important to note that our review does not address other forms of sexual violence, such as sexual harassment, female genital mutilation, forced marriage, or child sexual abuse.

Our systematic review aims to provide a synthesis of the literature on the barriers to the reporting, investigating, prosecuting, and sentencing of sexual assault and rape of adult women to inform future practice and policy for victim-survivors.

## Method

For our systematic review, we followed the Preferred Reporting Items for Systematic Reviews and Meta-Analyses (PRISMA-P) guidelines (Moher et al., 2015). Our study protocol was registered on the *Open Science Framework* (OSF) platform prior to conducting the review (https://osf.io/nza86/). Our search encompassed studies published until August 3, 2023, and involved collaboration with a librarian from the University of Western Australia to identify appropriate electronic databases, search terms, and operators. The following databases were searched: (a) PsycINFO, (b) MEDLINE, (c) Cochrane Library, (d) Scopus, (e) ProQuest Central, and (f) Web of Science. In addition, the gray literature sources (g) MedNar and (h) ProQuest Dissertations & Theses were also searched. For our search strategy, we combined relevant terms for the concept of sexual assault and rape (e.g., sexual assault, sexual violence, sexual victimization), barriers (e.g., disclos*, report*, attrition, and sentenc*), and gender (wom*n and femal*). For all eight databases, we used their option to filter the results for publications that have been published between January 2013 and August 2023. The results from the search were imported into *EndNote 20* (Clarivate) for further analysis and review.

### Eligibility Criteria

All published sources discussing barriers to the formal disclosure of rape and sexual assault to the criminal justice system, and/or discussing barriers to the investigation, prosecution, or conviction of rape and sexual assault were considered. Studies were included if they (a) provided qualitative or quantitative data on any factor(s) related to the decision not to formally disclose rape, including threatened, attempted, and completed vaginal, oral, or anal penetration, or sexual assault, including unwanted sexual touching with or without force, to the criminal justice system, (b) provided qualitative or quantitative data on any factor(s) related to the decision to disclose rape and/or sexual assault to support providers (e.g., healthcare providers, educational institutions) but not formally disclose to the criminal justice system, (c) provided qualitative or quantitative data on any factor(s) related to not investigate, prosecute, or convict rape, and/or sexual assault within any stage of the criminal justice system, (d) included female participants, regardless of race ethnicity, sexual orientation, gender identity, or disability status, who have experienced sexual victimization, (e) included participants who were 18 years or older, (f) took place in a Western country (i.e., United States, Canada, the European Union (including the UK), Norway, Iceland, Switzerland, Australia, or New Zealand), (g) were available in full text in English or German, and (h) had been published between January 1, 2013, and August 3, 2023.

Sources were excluded if they (a) focused on other forms of violence (e.g., physical, psychological, emotional, financial) or sexual or gender-based violence (e.g., sexual harassment, child sexual abuse, female genital mutilation, incest, stalking, and sexual exploitation), (b) focused on any form of sexual violence in the military or prison setting, (c) were based on a purely theoretical or conceptual approach, and (d) were unavailable for full-text review through institutional access and direct correspondence with the author (i.e., three email requests, separated by two weeks). Furthermore, we only included studies on interpersonal violence, family and domestic violence, or intimate partner violence if they specifically addressed sexual victimization. Studies that solely covered other forms of violence, such as physical, psychological, or emotional violence, were excluded during the full-text review.

### Selection Process

Two reviewers (MW and ST) independently conducted the screening of the retrieved literature. The title and abstract screening was performed using *Rayyan* in the first step. *Rayyan* is a web and mobile application designed to expedite the screening of abstracts and titles for systematic and meta-analyses. We used Kohen’s Cappa, which measures the agreement between two raters on items placed into mutually exclusive categories, to calculate the interrater reliability (Cohen, 1960). The interrater reliability between the reviewers was good (κ = .67; Cicchetti & Sparrow, 1981). In the second step, a full-text review was conducted using *Covidence* based on the predetermined eligibility criteria, and the interrater reliability was excellent (κ = .96; Cicchetti & Sparrow, 1981). *Covidence* is a web-based platform that simplifies the screening process when conducting literature reviews, including systematic reviews. In cases where consensus was not achieved, the suitability of the literature was discussed among the co-authors until a consensus was reached.

### Data Collection

Data from eligible studies were extracted and recorded, capturing predetermined information such as publication type (e.g., journal article, dissertation), study design, location, sample size, population, participant demographics, offense characteristics, and the barriers to reporting, investigating, prosecuting, and sentencing.

The data extraction process was conducted by a single author (MW). When the original authors explicitly defined and outlined barriers to any stage in the criminal justice system, we recorded these barriers using the terminology they provided. In instances where the terminology was unclear or intersecting, all co-authors decided on the preferred terminology, and overlapping categories were subsequently summarized into these broader categories. Examples of these broader barriers include (a) *self-blame, shame, and/or guilt*, (b) *fear of consequences (e.g., getting in trouble, getting others in trouble, losing someone, or concerns about personal safety)*, and (c) *minimization and/or denial of the experience*. These broader categories reflect recurring, overlapping barriers that were consistently discussed across publications with slight variations in wording.

### Deviations From Registered Protocol

During the screening of titles and abstracts, amendments were made to the inclusion and exclusion criteria initially preregistered in July 2023. Studies were excluded if they included male participants and reported barriers without specifying gender differences for male and female participants. Moreover, studies reporting barriers for victim-survivors who were underage at the time of sexual victimization were also excluded. This criterion extended to studies where participants were at least 18 years old during the study but were younger than 18 at the time of their sexual victimization. This adjustment was necessary because the initial search produced an extensive range of studies, leading to a lack of focus and cohesiveness in the review.

## Results

The initial search yielded 21,492 results. After the de-duplication in *EndNote 20* (Bramer et al., 2016), 8,655 articles were included for the title and abstract review. Out of these, 387 articles were suitable for the full-text review. The final review includes 28 articles, while 354 were excluded based on our criteria. Additionally, five duplicate articles were identified that were not previously detected by *EndNote 20*’s de-duplications process. For the exact search strategy and a list of excluded studies and the reasons for their exclusion, refer to the project’s OSF page (https://osf.io/nza86/). Supplemental material Appendix 1 provides an overview of the inclusion and exclusion process.

### Risk of Bias Analysis

The Mixed Methods Appraisal Tool (MMAT) Version 2018 was used to assess the quality of the 28 included studies (Hong et al., 2018). The MMAT is designed to appraise the methodological rigor of quantitative, qualitative, and mixed methods studies. For our studies, we used their qualitative research, quantitative descriptive studies, and mixed methods studies categories. Studies were assessed on five criteria reflective of their study type and each criterion was either coded “Yes,” “Can’t tell,” or “No.” As per the authors’ recommendation, we did not calculate scores or overall ratings from the criterion ratings.

Except for one study (Lindquist et al., 2016), which addressed nonresponse bias by weight adjustment, the descriptive quantitative and mixed methods studies were rated higher for their risk of nonresponse bias (Baker & Hassan, 2021; Carson et al., 2020; Cohn et al., 2013; Guidry et al., 2021; Johnson & Lewis, 2023; Jones, 2020; Kelley, 2023; Lamade et al., 2016; Tamarit Sumalla et al., 2023; Wood & Stichman, 2018). Two studies required additional information to assess their risk of nonresponse bias (Pijlman et al., 2023; Spencer et al., 2017). Nonresponse bias, defined as the difference between individuals who respond to a survey and those who do not, may lead to the inaccurate representation of the population in question and might affect the generalizability of the findings (Berg, 2005; Sax et al., 2003). Generally, individuals might decide not to participate in surveys addressing sexual assault and rape due to its sensitive nature, specifically if they have past experiences (Rosenberg et al., 2019), leading to potential differences between respondents and non-respondents. Prior research indicated that responses on sexual violence surveys across college differed in terms of demographics and the extent participants complete the surveys (Giroux et al., 2020). Moreover, several studies did not report how many respondents began their survey but did not complete it which would have further helped on assessing their risk of nonresponse bias (Carson et al., 2020; Pijlman et al., 2023; Spencer et al., 2017). Another methodological weakness was insufficient details regarding their sampling strategy and target population (Carson et al., 2020). In the Supplemental materials, Figures 1–3 show the summary plots of all three study categories (McGuinness & Higgins, 2020). Supplemental materials Figure 4 shows the appraisal of all included studies divided by qualitative, quantitative descriptive, and mixed methods design.

### Characteristics of Included Studies

Most sources were empirical research articles (*k* = 26) and two doctoral dissertations of which none were preregistered. Most of the studies used a qualitative research design (*k* = 15) with fewer using quantitative measures (*k* = 7) or a mixed methods design (*k* = 6). The total number of participants across all studies were *n* = 2,172, ranging from *n* = 5 to *n* = 441. The largest sample was based on women who have experienced rape and did not report it to law enforcement (*n* = 441; Cohn et al., 2013).

Study populations consisted either of women who were sexually victimized (*n* = 1,725; 79.42%) or criminal justice professionals (e.g., police officers, prosecutors) and key informants (e.g., victim advocates; *n* = 447; 20.58%) sharing insights. Additionally, three studies reviewed cases of female adult sexual victimization (*n* = 1,052) that have been reported to law enforcement (Murphy et al., 2014; O’Neal et al., 2015; Tamarit Sumalla et al., 2023). Furthermore, one study analyzed Tweets (*n* = 1,000) by women recounting their experiences of sexual victimization (Guidry et al., 2021).

For victim-survivors of sexual violence, only 9 out of the 15 studies provided demographic information beyond the gender. The demographics were reported for *n* = 1,023 individuals, leaving 702 participants with unclear details beyond identifying as women. Women who experienced sexual victimization identified themselves as predominantly White (*n* = 620; 62.69%), followed by Black (*n* = 266; 26.90%) and Hispanic (*n* = 127; 12.84%). Limited participation was observed from women who identified as American Indian or Alaska Native (*n* = 56; 5.66%), Asian (*n* = 10; 1.01%), Middle Eastern or North African (*n* = 2; 0.20%), and Native American/Other Pacific Islander (*n* = 1; 0.10%). Additionally, individuals of other ethnicities and races, apart from White, Black, or Hispanic, were often grouped under labels such as “other race” or similar classifications. The discrepancy in the number of individuals with reported demographics and provided ethnicities can be attributed to two studies instructing participants to declare all applicable ethnicities (Johnson & Lewis, 2023; Spencer et al., 2017). There was a limited representation of diverse gender identities, and except for one study, which included non-binary individuals (*n* = 5), all studies characterized their samples using binary categories such as women (*n* = 1,675) and men. Age ranged from 18 to 65 years. Mean and standard deviation for age could not be computed across studies due to a lack of detail.

For criminal justice professionals and key informants, only five (50%) out of 10 studies provided demographic information beyond the gender. The demographics were reported for *n* = 218 individuals (48.77%), leaving 229 participants (51.23%) with unclear details. The demographic breakdown of criminal justice professionals and key informants was predominantly White (*n* = 169; 94.94%), followed by Indigenous (*n* = 6; 3.37%), Hispanic (*n* = 2; 1.12%), Black (*n* = 1; 0.56%), and biracial (*n* = 1; 0.56%). Age ranged from 22 to 52 years. Again, mean and standard deviation for age could not be computed across studies due to a lack of detail. Criminal justice professionals and key informants were exclusively described using binary categories such as women (*n* = 200; 49.88%) and men (*n* = 201; 50.12%).

Barriers to formally reporting sexual victimization were most frequently researched across studies (*k* = 16) followed by barriers to investigating (*k* = 7), barriers to prosecuting (*k* = 5), and least frequently, barriers to sentencing (*k* = 2). One study reported on both barriers to investigating and prosecuting (Murphy et al., 2014). Only a small number of studies reported details regarding the offense and perpetrator (*k* = 5). Most studies have been conducted in the United States (*k* = 21), followed by the United Kingdom (*k* = 3), Canada (*k* = 2), Spain (*k* = 1), Sweden (*k* = 1), and the Netherlands (*k* = 1). Study populations consisted of individuals working in the criminal justice system (e.g., police officers and prosecutor; *k* = 10), student samples (*k* = 9), adult women (*k* = 4), Twitter users (*k* = 2), and female patients and medical personnel (*k* = 1). Three studies reviewed cases of sexual violence (*n* = 1,052) which offered insights into prosecutorial decision-making. The sections on barriers to reporting and investigating focus on the most prominently discussed obstacles in the literature for conciseness. A full list of the studies and their reported barriers is found in Table 1 reporting the critical findings for all included studies.

**Table 1.** Critical Findings of Included Studies.

First Author	Country	Sample	Main Findings (Barriers Associated With): (a) Reporting (b) Investigating (c) Prosecuting (d) Sentencing
Annan (2014)	US	18 prosecutors, sheriffs, and victim-witness advocates	(c) Lack of DNA evidence; Jurors increasingly less convinced by testimonial evidence; Jurors want near certainty; Lack of physical evidence and/or other corroborating evidence (e.g., witnesses); Victim does not want to prosecute; Memory issues hampering testimony; Forensic nurses inexperienced in giving effective
Baker and Hassan (2021)	US	143 prosecutors and 96,914 arrest charges which require an Assistant District Attorney to accept the charge for prosecution or reject the charge	(c) *Female prosecutor with limited experience; Male prosecutor*
Caron and Mitchell (2022)	US	Fourteen college women who had never shared their adult sexual assault with anyone prior to speaking to the researchers	(a) To avoid stigma and/or label; Self-blame, shame, and/or guilt; Fear of consequences, for example, getting in trouble, getting others in trouble, losing someone, or own safety; Fear others will blame/humiliate me; *Wanting to pretend it never happened/move forward; Fear of losing control of situation/too much trouble*; Fear of not being believed
Carson et al. (2020)	US	Fifty-six undergraduate women who experienced sexual victimization	(a) Self-blame, shame, and/or guilt; Fear of consequences, for example, getting in trouble, getting others in trouble, losing someone, or own safety; Minimization and/or denial of the experience; *Need for privacy/confidentiality*
Cohn et al. (2013)	US	Four hundred forty-one women who had experienced a most recent or only rape incident and did not report the incident to the police	(a) Fear of consequences, for example, getting in trouble, getting others in trouble, losing someone, or own safety; Minimization and/or denial of the experience; *Need for privacy/confidentiality*; Lack of supporting evidence that incident happened; Fear of experiencing mistreatment by law enforcement, legal professionals, or other components of the justice system; Did not know how and/or where to report
Gekoski et al. (2024)	UK	Seventeen police officers	(b) Pre-existing bias that victims fabricate their experiences of sexual victimization; *Pre-existing bias that victims do omit important details; Victims consistently making multiple false allegations over time*; Prior consensual sexual contact with perpetrator; Being a sex worker; Having a mental illness; Intoxication at the time of offense
Guidry et al. (2021)	US	One thousand Tweets by female Twitter users	(a) Self-blame, shame, and/or guilt; Fear of consequences, for example, getting in trouble, getting others in trouble, losing someone, or own safety; Fear others will blame/humiliate me; *Wanting to pretend it never happened/move forward*; Fear of not being believed; Minimization and/or denial of the experience; Fear of experiencing mistreatment by law enforcement, legal professionals, or other components of the justice system; *Negative reaction after informal disclosure; Social status of perpetrator; Threatened*; Lack of Legal Accountability for the Perpetrator by CJS; *Law enforcement and/or university did not take action in past; Perpetrator is/was in law enforcement; Fear*
Henry (2020)	US	Eight Black female college students who experienced sexual assault between 1989 and 1999	(a) To avoid stigma and/or label; Self-blame, shame, and/or guilt; Fear of consequences, for example, getting in trouble, getting others in trouble, losing someone, or own safety; Fear others will blame/humiliate me; *Wanting to pretend it never happened/move forward ; Fear of losing control of situation/too much trouble*; Fear of not being believed; Minimization and/or denial of the experience; Fear of experiencing mistreatment by law enforcement, legal professionals, or other components of the justice system; Did not know how and/or where to report; *Negative reaction after informal disclosure; Social status of perpetrator; Law enforcement and/or university did not take action in past; Fear; Feelings of being insignificant; (Previous) sexual victimization and silence is norm; Unclear or non-existent college policies on sexual assault*
Huemmer et al. (2019)	US	Five female students who never reported their rape	(a) To avoid stigma and/or label; Self-blame, shame, and/or guilt; Fear others will blame/humiliate me; *Fear of losing control of situation/too much trouble*; Lack of Legal Accountability for the Perpetrator by CJS; *Perpetrator is/was in law enforcement*
Johnson and Lewis (2023)	US	Ninety-four women and non-binary	(a) To avoid stigma and/or label; Self-blame, shame, and/or guilt; Fear of consequences, for example, getting in trouble, getting others in trouble, losing someone, or own safety; *Wanting to pretend it never happened/move forward; Fear of losing control of situation/too much trouble* ; Fear of not being believed; Minimization and/or denial of the experience; *Need for privacy/confidentiality*; Lack of supporting evidence that incident happened; Fear of experiencing mistreatment by law enforcement, legal professionals, or other components of the justice system; Lack of Legal Accountability for the Perpetrator by CJS; *Law enforcement and/or university did not take action in past; (Previous) sexual victimization and silence is norm; Did not want to travel/accessibility difficulties; Relationship to perpetrator; Suspect and/or victim were intoxicated by alcohol and/or other substances; Disclosed to someone other than police; Somebody in my family dealt with it*
Jones (2020)	US	Twenty female students who reported an unwanted sexual experience while enrolled in college and agreed to a semi-structured interview and did not report to law enforcement	(a) Self-blame, shame, and/or guilt; Fear of consequences, for example, getting in trouble, getting others in trouble, losing someone, or own safety; Fear others will blame/humiliate me; Fear of not being believed; Minimization and/or denial of the experience; Lack of supporting evidence that incident happened; Fear of experiencing mistreatment by law enforcement, legal professionals, or other components of the justice system; *Negative reaction after informal disclosure; Social status of perpetrator*
Kelley (2023)	US	One hundred seventy-two Black women	(a) *Suspect and/or victim were intoxicated by alcohol and/or other substances; Experienced bleeding or were unsure of bleeding in the vaginal area after rape/assault*
Lamade et al. (2016)	US	One hundred thirty undergraduate women who reported yes to “Someone had sex with me at a party after I had too much to drink, but I did not report it”	(a) To avoid stigma and/or label; Self-blame, shame, and/or guilt; Fear of consequences, for example, getting in trouble, getting others in trouble, losing someone, or own safety; Fear others will blame/humiliate me; Minimization and/or denial of the experience; *Need for privacy/confidentiality*; Lack of supporting evidence that incident happened; Did not know how and/or where to report; Lack of Legal Accountability for the Perpetrator by CJS; *Law enforcement and/or university did not take action in past; Relationship to perpetrator; Too anxious/too depressed*
Lindquist et al. (2016)	US	Four hundred thirty-eight undergraduate female who had experienced sexual assault since entering college	(a) Self-blame, shame, and/or guilt; Fear of consequences, for example, getting in trouble, getting others in trouble, losing someone, or own safety; Minimization and/or denial of the experience; *Need for privacy/confidentiality*; Lack of supporting evidence that incident happened; Fear of experiencing mistreatment by law enforcement, legal professionals, or other components of the justice system; Did not know how and/or where to report; Lack of Legal Accountability for the Perpetrator by CJS; *Relationship to perpetrator; Suspect and/or victim were intoxicated by alcohol and/or other substances; Did not think a female officer or officer of the same race/ethnicity would be available*
McMillan (2018)	UK	Forty police officers	(b) Having a mental illness; Intoxication at the time of offense; Lack of cooperation and/or withdrawing complaint altogether; Inconsistencies in the complaint; Pre-existing bias that women use false complaints to seek attention and/or revenge; *Pre-existing bias that women use false complaints to cover consensual sexual encounters*; Lack of physical evidence and/or other corroborating evidence (e.g., witnesses)
Murphy et al. (2013)	US	Law enforcement (*n* = 8), prosecutors (*n* = 8), victim witness advocates (*n* = 10), sexual assault nurse examiners (*n* = 11), and crisis center advocates (*n* = 14)	(c) Lack of DNA evidence; Jurors want near certainty; Lack of physical evidence and/or other corroborating evidence (e.g., witnesses); Impact of delayed reporting on juror perception of incident; “Beyond reasonable doubt” standard very high for jurors to find guilty verdict
Murphy et al. (2014)	US	Forty one female sexual assault cases in which police specifically reported that victims chose to not pursue their case	(b) Lack of cooperation and/or withdrawing complaint altogether(c) Victim did not want to prosecute after initial complaint was made “(chose to drop the case”)
O’Neal et al. (2015)	US	Forty-seven intimate partner sexual assault complaints	(c) Lack of DNA evidence; Lack of physical evidence and/or other corroborating evidence (e.g., witnesses); Victim does not want to prosecute; Victim not credible; No additional domestic violence factors (e.g., stalking, controlling behavior)
Perry et al. (2015)	US	Female patients (*n* = 9), abortion providers (*n* = 5), rape victim advocates (*n* = 4), social workers (*n* = 2), and clinic administrators at abortion clinics (*n* = 1)	(a) Fear of not being believed; Lack of supporting evidence that incident happened; *Social status of perpetrator*; Lack of Legal Accountability for the Perpetrator by CJS; *Law enforcement and/or university did not take action in past*
Pijlman et al. (2023)	Netherlands	Forty-five non-help seeking women after experiencing sexual violence	(a) To avoid stigma and/or label; Fear of consequences, for example, getting in trouble, getting others in trouble, losing someone, or own safety; Minimization and/or denial of the experience; Fear of experiencing mistreatment by law enforcement, legal professionals, or other components of the justice system; *Did not want to travel/accessibility difficulties*
Ricciardelli et al. (2021)	Canada	Seventy police officers in sex crime-related units	(b) Being a sex worker; Intoxication at the time of offense; Lack of physical evidence and/or other corroborating evidence (e.g., witnesses); *Rape myths/Victim blaming*
Rudolfsson (2022)	Sweden	Sixteen police officers	(b) *Prosecutors prematurely closing cases officers wished to further investigate*
Sinclair (2022)	UK	Twenty-nine active or retired officers who had experience of working on rape cases	(b) Being a sex worker; Intoxication at the time of offense; Inconsistencies in the complaint; *Delayed reporting; Age (i.e., younger less credible); Perpetrator*’*s credibility deemed higher than victim*’*s credibility; Any behavior prior to the offense that is deemed to suggest consent on victim*’*s side; Rape myths/Victim blaming; Jury unlikely to convict; Selective prioritization: Allocating investigative resources to stronger cases over weaker cases; Variability in investigation quality stemming from resource and staff constraints*
Spencer et al. (2017)	US	Two hundred twenty female students who reported that they had “experienced any sort of nonconsensual or unwanted sexual contact”	(a) Self-blame, shame, and/or guilt; Fear of consequences, for example, getting in trouble, getting others in trouble, losing someone, or own safety; Fear others will blame/humiliate me; Minimization and/or denial of the experience; Did not know how and/or where to report; *Fear*
Spencer et al. (2018)	Canada	Seventy police officers in sex crime-related units	(b) Pre-existing bias that victims fabricate their experiences of sexual victimization; Inconsistencies in the complaint; Pre-existing bias that women use false complaints to seek attention and/or revenge (d) Judicial actors lack training in understanding complexity and impact of sexual assault cases; Court system not compatible with taking victims’, witnesses, and families’ needs into account during the court process
Tamarit Sumalla et al. (2023)	Spain	Nine hundred sixty-four cases involving adult women that had filed a charge for a crime of rape as a case.	(d) Perpetrator being current partner; Perpetrator being ex-partner
Venema (2016)	US	Ten police officers	(b) Pre-existing bias that victims fabricate their experiences of sexual victimization; Prior consensual sexual contact with perpetrator; Being a sex worker; Inconsistencies in the complaint; Pre-existing bias that women use false complaints to seek attention and/or revenge; Lack of physical evidence and/or other corroborating evidence (e.g., witnesses); *Victim demeanor lacks emotion during reporting*
Wood and Stichman (2018)	US	Seventy-three female students who answered additional questions about the circumstances of the assault or coercive event and noted whether or not they reported it formally or informally	(a) Self-blame, shame, and/or guilt; Fear of consequences, for example, getting in trouble, getting others in trouble, losing someone, or own safety; Fear of not being believed; Minimization and/or denial of the experience; *Need for privacy/confidentiality*; Fear of experiencing mistreatment by law enforcement, legal professionals, or other components of the justice system; Did not know how and/or where to report; *Negative reaction after informal disclosure*; Lack of Legal Accountability for the Perpetrator by CJS; *Law enforcement and/or university did not take action in past*

*Note.* Italicized barriers are not discussed in the results section on reporting and investigating.

### Barriers to Reporting

The reviewed articles indicate a wide range of barriers to formally reporting sexual violence. In total, we found 30 unique barriers reported across 15 studies. The most frequent barriers to formally report sexual violence were *fear of consequences, for example, getting in trouble, getting others in trouble, losing someone, or own safety* (*k* = 12), followed by *self-blame, shame, and/or guilt* (*k* = 11), and *minimization and/or denial of the experience* (*k* = 11). Moreover, *fear of experiencing mistreatment by law enforcement, legal professionals, or other components of the justice system* (*k* = 8), *fear others will blame/humiliate me* (*k* = 7), *fear of not being believed* (*k* = 7), *lack of legal accountability for the perpetrator by criminal justice system* (*k* = 7), *did not know how and/or where to report* (*k* = 6), *to avoid the stigma or label (k* = 6), and the *lack of supporting evidence that the incident happened (k* = 6) were commonly reported across studies. These findings collectively emphasize the intricate nature of barriers to reporting, covering psychosocial, procedural, and societal challenges.

Only three studies reported quantifiable details for assault characteristics and victim–perpetrator relationship. Three studies had participants who experienced a drug-facilitated sexual assault (*n* = 122, 23.87%; Caron & Mitchell, 2022; Carson et al., 2020; Cohn et al., 2013), and two studies had participants who experienced penetration (*n* = 389, 85.49%; Caron & Mitchell, 2022; Cohn et al., 2013). Most victim-survivors reported to have been victimized by an acquaintance, friend, or relative (*n* = 267, 58.68%), an intimate or ex-intimate partner (*n* = 116, 25.49%), and by a stranger (*n* = 41, 9.01%). The majority of incidents involved a lone perpetrator (*n* = 13, 92.85%) who identified as male (*n* = 12, 85.71%) and was known to them (*n* = 13, 92.85%).

### Barriers to Investigating

In total, we found 22 unique barriers to investigating formal reports of sexual victimization across 8 studies. All eight included police officers in their study population. The most frequent barrier to investigating complaints of sexual victimization were *intoxication at the time of the offense* (*k* = 4), *being a sex worker* (*k* = 4), *pre-existing bias that victims fabricate their experiences of sexual victimization* (*k* = 4), *inconsistencies in the complaint* (*k* = 4), *pre-existing bias that women use false complaints to seek attention and/or revenge* (*k* = 3), and *lack of physical evidence and/or other corroborating evidence (e.g., witnesses; k* = 3). These barriers can manifest as skepticism or disbelief in the police officers, with the perception that complainants might fabricate their allegations ant that intoxicated complainants and sex workers lack credibility. Moreover, the presence of inconsistencies and the lack of concrete evidence might cast doubt on the strength of the case for further investigation and forwarding it to the prosecution.

Other barriers to investigating were prior consensual sexual contact with perpetrator (*k* = 2), having a mental illness (*k* = 2), and lack of cooperation and/or withdrawing complaint altogether (*k* = 2). Similarly, these barriers might impact the perceived credibility of the complainant, their account, and the perceived strength of the case for laying charges and the likelihood of a conviction by the judge or jury. Overall, these barriers should be viewed as interlinked factors, not isolated barriers, that impede the investigation and investigation efforts. We were able to extract further details on the perpetrator from one study. Most perpetrators were known to the victim-survivor (*n* = 38, 93.00%) with the majority being acquaintances or relatives (*n* = 24, 57.50%) and all of them acting alone and being male (*n* = 41, 100%).

### Barriers to Prosecuting

In total, we found 14 unique barriers to prosecuting complaints of sexual victimization across six studies. The most frequent barriers were *lack of DNA evidence* (*k* = 3), *lack of physical evidence and/or other corroborating evidence (e.g., witnesses; k* = 2), *jurors want near certainty* (*k* = 2), and *victim not credible* (*k* = 2). Other barriers that were mentioned once were *jurors increasingly less convinced by testimonial evidence, victim does not want to prosecute, memory issues hampering testimony, forensic nurses inexperienced in giving effective testimony, female prosecutor with limited experience, male prosecutor, impact of delayed reporting on juror perception of incident, “beyond reasonable doubt” standard very high for jurors to find guilty verdict, victim did not want to prosecute after initial complaint was made (“chose to drop the case”)*, and *no additional domestic violence factors (e.g., stalking, controlling behavior)*. Each of these factors may affect the perceived strength of the case and the viability to achieve a conviction by a judge or jurors. The same single study for barriers to investigate which reported details on the perpetrator also reported barriers to prosecuting. As previously mentioned, most perpetrators were known to the victim-survivor (*n* = 38, 93.00%) with the majority being acquaintances or relatives (*n* = 24, 57.50%) and all of them acting alone and being male (*n* = 41, 100%).

### Barriers to Sentencing

We found four barriers to sentencing cases of sexual victimization across two studies. When the *perpetrator is the current partner* (*k* = 1) or *the ex-partner* (*k* = 1) judges seem more reluctant to impose adequate sentences reflecting the gravity of the offense. Moreover, the *judicial actors lack training in understanding complexity and impact of sexual assault cases* (*k* = 1) and the *court system not being compatible with taking victims*’, *witnesses, and families’ needs into account during the court process* (*k* = 1) which limits their understanding of victim-survivors’ needs throughout the court process, might result in unjust decisions, and secondary victimization of victim-survivors.

## Discussion

To the best of our knowledge, this study represents the first attempt to synthesize barriers related to the stages of reporting, investigating, prosecuting, and sentencing of cases of sexual victimization in adult women in Western countries. Our analysis encompassed 28 studies, uncovering a total of 70 reported barriers.

Similar to previous studies (Orchowski et al., 2022; Stewart et al., 2023), overarching themes across all barriers and specifically the reporting stage might be summarized under lack of trust in the criminal justice system (e.g., *fear of not being believed, fear of experiencing mistreatment by law enforcement, legal professionals, or other components of the justice system*, and *lack of legal accountability for the perpetrator by criminal justice system*), internal reactions (e.g., *self-blame, shame, and/or guilt*, and *fear;* Weiss, 2010), rape myths and societal norms (e.g., *minimization and/or denial of the experience, intoxication at the time of offense*, and *pre-existing bias that women use false complaints to seek attention and/or revenge*), and perpetrator characteristics (e.g., *social status of perpetrator, perpetrator’s credibility deemed higher than victim’s credibility*, and *perpetrator being current partner or ex-partner*). These overarching themes and barriers portray a complex decision-making process wherein survivors carefully consider various societal, contextual, interpersonal, and intrapersonal factors before deciding whether to report. The consistency in themes across studies and past findings offers some validation that major barriers to reporting have been covered. Building on our findings and the existing literature, these recurring themes can be more comprehensively understood when viewed as distinct manifestations of stigma (Kennedy & Prock, 2018).

Most impediments to the investigation stage of sexual victimization revolve around the credibility of the victim-survivor. Instances where the victim-survivor was intoxicated during the offense are frequently seen as undermining the victim-survivor’s credibility and may be perceived as a risky behavior on the victim-survivor’s part (Gekoski et al., 2024; McMillan, 2018; Ricciardelli et al., 2021; Sinclair, 2022). Consequently, these circumstances may result in a reduced commitment to the investigation (Sinclair, 2022). Paradoxically, in cases where the level of intoxication is so extreme that the victim-survivor would have been incapable of providing consent, it can benefit the investigation. As one officer noted, “Sometimes the levels of intoxication assists your case, due to once you get toxicology results, it kind of goes to the point where that person is that intoxicated that there’s no way they could have even consented.” (Gekoski et al., 2024, p. 13). A recent meta-analysis on factors impacting police decision-making indicated a significant reduction in arrest likelihood when the victim consumed alcohol prior or during the sexual assault (Lapsey et al., 2022).

Similarly, the credibility of individuals with a mental illness (Gekoski et al., 2024; McMillan, 2018) or who are engaging in sex work (Gekoski et al., 2024; Ricciardelli et al., 2021; Sinclair, 2022; Venema, 2016) is often scrutinized. As one officer noted, “I’ve never known a case where the victim-survivor’s place in society makes any difference, except where the female is a prostitute and is raped.. . . Which is not fair as no one should be judged on what they do [. . .].” (Sinclair, 2022, p. 145). This inappropriate emphasis on victim-survivor credibility has been acknowledged among officers, with one noting, “We massively pre-judge the credibility of the victim. We investigate the victim more than the offence itself” (Gekoski et al., 2024, p. 8).

The repercussions of perceived low victim-survivor credibility are profound. Officers have described “ambiguous” cases which involve intoxication, the victim-survivors having a prior or current relationship to the perpetrator, or the victim-survivor and the perpetrator being acquainted (Gekoski et al., 2024; Venema, 2016). These cases lack supporting evidence, yet the occurrence of sexual victimization is not considered false. Credibility of the victim-survivor has previously been found to affect the amount of investigative effort and the likelihood of referring the case for charging (Campbell et al., 2015; Sinclair, 2022). A meta-analysis by Lapsey et al. (2022) supports this, showing decreased arrest likelihood in cases of sexual assault when factors impacting victim credibility were known to police officers. Overall, the scrutiny of victim-survivor credibility at the investigation stage can have significant consequences for the trajectory of justice in sexual victimization cases.

Police officers have expressed that the stringent standard of proving guilt beyond a reasonable doubt (Ricciardelli et al., 2021; Venema, 2016) and the assumption that juries are unlikely to convict (Sinclair, 2022) serve as barriers to further investigating. However, this pre-emptive mindset about juries and trials may be influenced more by the officers’ own victim-blaming perspectives rather than the actual views of the juries (Sinclair, 2022). Together with previous studies indicating that most attrition happens at the police investigation stage (Hester & Lilley, 2017; Spohn & Tellis, 2012), our results reinforce the idea that police officers can function as gatekeepers to justice (Frazier & Haney, 1996) with police officers scrutinizing victim-survivors of sexual violence for factors beyond the victim-survivor’s control.

Our review indicates a limited number of studies focusing on the barriers influencing the prosecutorial and sentencing stages in cases of adult sexual victimization (Annan, 2014; Baker & Hassan, 2021; Murphy et al., 2014; O’Neal, 2016; Spencer et al., 2018; Tamarit Sumalla et al., 2023). Although extensive research has been conducted on the judgments of mock jurors and mock juries in sexual assault and rape cases (for a review, see Leverick, 2020), accessing the deliberation and decision-making of juries is prohibited by law in most Western countries with an adversarial legal system (e.g., Contempt of Court Act, 1981; Criminal Code, 1985). Additionally, engaging or recruiting judges for research purposes is notoriously difficult (Sporer & Goodman-Delahunty, 2009). Consequently, studies on sentencing have largely focused on reviewing court transcripts (Quilter et al., 2023) or sentencing remarks which can be further impacted by privacy concerns (Sporer & Goodman-Delahunty, 2009). Our findings on barriers to sentencing reflect this: authors either reviewed publicly available court sentences (Tamarit Sumalla et al., 2023) or cited police officers as they reflected on the shortcomings of the criminal justice system (Spencer et al., 2018). Future research may benefit from further exploring the factors that impact the decision-making of prosecutors and judges and gaining a comprehensive understanding of the high attrition rates throughout the criminal justice system (Daly & Bouhours, 2010). Such exploration is crucial, considering that reporting sexual violence to authorities marks the initial step, but numerous cases face dismissal during subsequent legal processes (Alderden & Ullman, 2012; Bright et al., 2021; Hohl & Stanko, 2015).

Collectively, the decreasing volume of studies conducted at each stage underscores the persistent attrition observed in rape cases within the criminal justice system across the globe (Daly & Bouhours, 2010; Hester & Lilley, 2017; Hohl & Stanko, 2015). The initial choice not to report to authorities emerges as a common source for attrition evidenced by a variety of reasons for victim-survivors not to report, succeeded by successive stages, namely the police investigation, prosecution, and sentencing phases (George & Ferguson, 2021; Rotenberg, 2017). In addition, conviction rates in sexual violence cases are low (Alderden & Ullman, 2012; Australian Bureau of Statistics, 2021; Bright et al., 2021; The Crown Prosecution Service, 2023).

Our review highlights that addressing the criminal justice system’s response to rape victim-survivors continues to pose substantial challenges, with the need for future development evident in the preconceptions and attitudes of police officers. While acknowledging that police officers are not the sole contributors to challenges faced by victim-survivors of rape and sexual assault in the criminal justice system, their response and treatment of victim-survivors reflect societal moral boundaries by delineating what behavior is and is not tolerated. Therefore, any changes in the police response could be a catalyst for transformative change in the quest to end sexual violence against women (Hohl & Stanko, 2022). Aside from this, it is imperative for future studies to explore ways to enhance the prosecuting and sentencing stages for victim-survivors. Additionally, exploring whether these barriers equally affect other subgroups of women, such as culturally and linguistically diverse (CALD) individuals and women with disabilities, is desirable.

To foster trust in the criminal justice system, governments should actively engage both victim-survivors and key players within the system. Ultimately, ensuring that individuals who experience sexual victimization are treated fairly and with due diligence, while providing criminal justice professionals with the necessary training and resources should be the goal. Several governments are already taking steps to address the shortcoming of their criminal justice system’s response to rape and sexual assault victim-survivors. In Australia, states are reviewing their criminal justice system responses to sexual violence to ensure that victim-survivors are supported and receive a justifiable outcome (Attorney-General’s Department, n.d.; Commissioner for Victims of Crime, 2023). Similarly, England and Wales have initiated *Operation Soteria*, a collaboration between academics and the police, aiming to transform the nation’s police forces response to rape (Stanko, 2022). Moreover, specialist training for police officers engaging with victim-survivors of sexual violence has demonstrated positive effects on their rape myth acceptance, perceived victim responsibility, and case progression (for a review, see McQueen & Murphy-Oikonen, 2023). However, shortcomings of the current evidence base include lack of power analyses, reliance on self-report measures, and few studies with a control group, limiting the generalization of these findings. Additionally, future research should focus on the optimal length, modality, and content of training. In conclusion, governments and police forces must address the current shortcomings in their treatment of victim-survivors of sexual violence.

### Limitations and Implications for Future Research

Although our systematic review offers theoretical and policy implications, it is not without limitations. Our search strategy as well as the decision to exclude studies that reported barriers without specifying gender differences for male and female participants and studies reporting barriers for victim-survivors who were underage at the time of sexual victimization has limited our scope. It allowed us to provide a very focused review of the barriers affecting adult women who have experienced sexual violence in Western countries. However, our review does not reflect the full spectrum of barriers victim-survivors are facing in the criminal justice system.

Our geographical focus allowed for a deeper, more nuanced understanding of the barriers to sexual assault and rape case progression within the criminal justice system in the context of Western countries. However, the generalizability of our findings to other regions with different legal, cultural, and social contexts requires further investigation. The global issue of underreporting sexual violence (World Health Organization, 2021) suggests a potential overlap with our identified barriers on a broader scale.

The reviewed empirical base was limited in the heterogeneity of sample demographics, eligibility criteria, and outcome measures. Hence, a meta-analysis was not possible. Furthermore, there was a lack of detail in reported sample demographics, type of sexual victimization, and offense characteristics. The studies that were reviewed primarily adopted a qualitative, exploratory, or descriptive approach, employing convenience or purposive sampling methods. As a result, our findings might reflect the inherent selection bias in the reported barriers.

The sample demographics of victim-survivors of sexual victimization also exhibited a notable limitation in diversity. Most samples consisted of White women and a restricted representation of various gender identities. However, this observation is based on the limited details that were reported, leaving the actual demographics in terms of ethnicity and gender identity unclear. The limited demographic information also restricts our ability to generalize findings to other subpopulations, such as CALD women and women with disabilities.

Similar to the limitations in the victim-survivor samples, the reviewed studies lacked demographic information for criminal justice professionals and key informants with only half providing information beyond gender. A lack of diversity across ethnic and racial groups was notable with the majority of participants identifying as White. Gender was exclusively reported using binary gender categories overlooking diverse gender identities. The limited demographic data restricts our understanding of the diversity within the criminal justice system, which might be a contributing factor to the negative experiences reported by victim-survivors. Prior research suggests that gender bias can influence police decision-making with male police officers placing less credibility on victims and showing higher rape myth acceptance than female police officers (for a review, see Sleath & Bull, 2017). Likewise, men in the general population demonstrate higher rape myth acceptance than women (e.g., Grubb & Turner, 2012). Gender statistics on police and judicial positions indicate a skewed gender distribution. Men frequently hold a majority in these positions, while women remain underrepresented (Australasian Institute of Judicial Administration Inc., 2023; Government Of Canada, 2024; Ministry of Justice, 2023; Statista, 2021). Future studies should explore how a more representative police and judicial workforce might impact decision-making in sexual assault and rape cases.

Most studies that were retained for review lacked details regarding the offense and perpetrator characteristics. Similarly, many studies did not specify how many participants reported each identified barrier. Barriers might vary depending on contextual factors, for instance the perpetrator being acquainted with the victim-survivor, which applies to many reported cases (Basile et al., 2022). These details might have provided a more nuanced understanding of the identified barriers. Ultimately, these methodological differences complicated the synthesis of our findings and highlighted the need for consistency in reporting practices across studies.

An additional limitation arose from the interchangeable use of terms such as sexual assault and sexual abuse, which often depict a spectrum of sexual offenses with varying interpretations depending on location. For example, in England and Wales, sexual assault specifically refers to the criminal act of unwanted sexual touching in police-recorded crimes (The Crown Prosecution Service, n.d.), yet public surveys may use the term more broadly including rape, unwanted touching, or indecent exposure (Office for National Statistics (ONS), 2023). Similarly, in the United States, sexual assault encompasses a wide range of sexual offenses (Office on Violence Against Women, 2022). To maintain focus in our findings, we had to omit studies exploring forms of sexual victimization beyond those aligned with our defined inclusion criteria.

Despite high rates of sexual victimization among women, barriers were frequently explored through open-ended questions, leading to a varied list of reasons why women chose not to report. We attempted to mitigate this variability by condensing frequently mentioned barriers into overarching categories. However, the diverse wording and themes made it more challenging to effectively summarize the barriers while ensuring that the list comprehensively represents our findings. Future research on barriers to reporting sexual victimization would benefit from establishing a tool that consistently measures reasons why victim-survivors decide not to report. Table 2 summarizes the main discoveries from our review along with their corresponding implications.

**Table 2.** Implications for Practice, Policy, and Research.

Practice	• Provide evidence-based specialist training and knowledge to lawyers, police, judiciary, and individuals who will be engaging with victim-survivors and cases of sexual assault and rape. • Improve transparency within the criminal justice system and its processes to foster trust and accountability by engaging and collaborating with victim-survivors and key informants. • Address attrition rates at each stage of the criminal justice system by implementing suspect-focused investigations and trauma-informed victim-centered support throughout the proceedings.
Policy	• Advocate for comprehensive reforms within the criminal justice system to address the identified barriers to reporting, investigating, prosecuting, and sentencing of sexual assault and rape by mandating evidence-based specialist training and trauma-informed interviewing techniques to police officers and aiming for a representative, diverse workforce in terms of gender and ethnicity. • Formulate and support legislative changes that promote victim-survivors’ needs and well-being by providing dedicated support services throughout proceedings, easing navigation through the criminal justice system, and improving overall outcomes of sexual assault and rape investigations.
Research	• Conduct research that actively engages with diverse populations to ensure all victim-survivors’ needs are known and can be adequately addressed. • Broaden the evidence base at each stage of the criminal justice system, specifically the sentencing stage, to allow effective and evidence-based information for policy and practice.

## Conclusion

In conclusion, the identified barriers to the reporting, investigating, prosecuting, and sentencing of sexual assault and rape against adult women underscore the critical imperative for reform within the criminal justice system. Central to this reform is prioritizing victim-survivors’ needs, improving the transparency of the criminal justice system and its processes, and addressing attrition rates. The attrition of reported cases throughout the criminal justice system was mirrored in the evidence base for each stage, with progressively fewer studies available from the reporting to the sentencing stage. Future studies need to focus on greater engagement with diverse populations to ensure that all victim-survivors’ needs can be addressed. Moreover, gaining further insights on the challenges at all stages of the criminal justice system for improving the treatment of victim-survivors and enhancing the overall effectiveness of rape and sexual assault investigations is needed.

## Supplemental Material

sj-docx-1-tva-10.1177_15248380241261404 – Supplemental material for Silenced Survivors: A Systematic Review of the Barriers to Reporting, Investigating, Prosecuting, and Sentencing of Adult Female Rape and Sexual Assault Supplemental material, sj-docx-1-tva-10.1177_15248380241261404 for Silenced Survivors: A Systematic Review of the Barriers to Reporting, Investigating, Prosecuting, and Sentencing of Adult Female Rape and Sexual Assault by Michelle Wieberneit, Sascha Thal, Joseph Clare, Lies Notebaert and Hilde Tubex in Trauma, Violence, & Abuse
