# Supplementary material for: Silenced Survivors: A Systematic Review of the Barriers to Reporting, Investigating, Prosecuting, and Sentencing of Adult Female Rape and Sexual Assault
Source: Trauma Violence Abuse. 2024 Jul 30;25(5):3742–57. doi: 10.1177/15248380241261404 (PMC11545439; doi:10.1177/15248380241261404)
Supplement: sj-docx-1-tva-10.1177_15248380241261404 – Supplemental material for Silenced Survivors: A Systematic Review of the Barriers to Reporting, Investigating, Prosecuting, and Sentencing of Adult Female Rape and Sexual Assault [file sj-docx-1-tva-10.1177_15248380241261404.docx]

**Appendix 1**

PRISMA Flowchart

**Identification of studies via databases and registers**

**Identification of studies via databases and registers**

Records identified from:

PsycINFO (n = 2539)

Medline (n = 4908)

Cochrane (n = 195)

Scopus (n = 6959)

ProQuest Central (n = 1893)

Web of Science (n = 4807)

Mednar (n = 167)

ProQuest Theses (n = 24)

Records removed *before screening*:

Duplicate records removed (n = 12837)

**Identification**

Records screened:

(n = 8655)

Records excluded:

(n = 8268)

Reports sought for retrieval:

(n = 387)

Reports not retrieved:

(n = 0)

**Screening**

Reports assessed for eligibility:

(n = 382)

Reports excluded: (n = 354)

Studies included in review

(n = 28)

**Included**

*Note.* Adapted from Page, M. J., McKenzie, J. E., Bossuyt, P. M., Boutron, I., Hoffmann, T. C., Mulrow, C. D., ... Moher, D. (2021). The PRISMA 2020 statement: An updated guideline for reporting systematic reviews. *bmj, 372*, n71. https://doi.org/10.1136/bmj.n71

**Figure 1**

*Qualitative Research Studies Summary Plot*


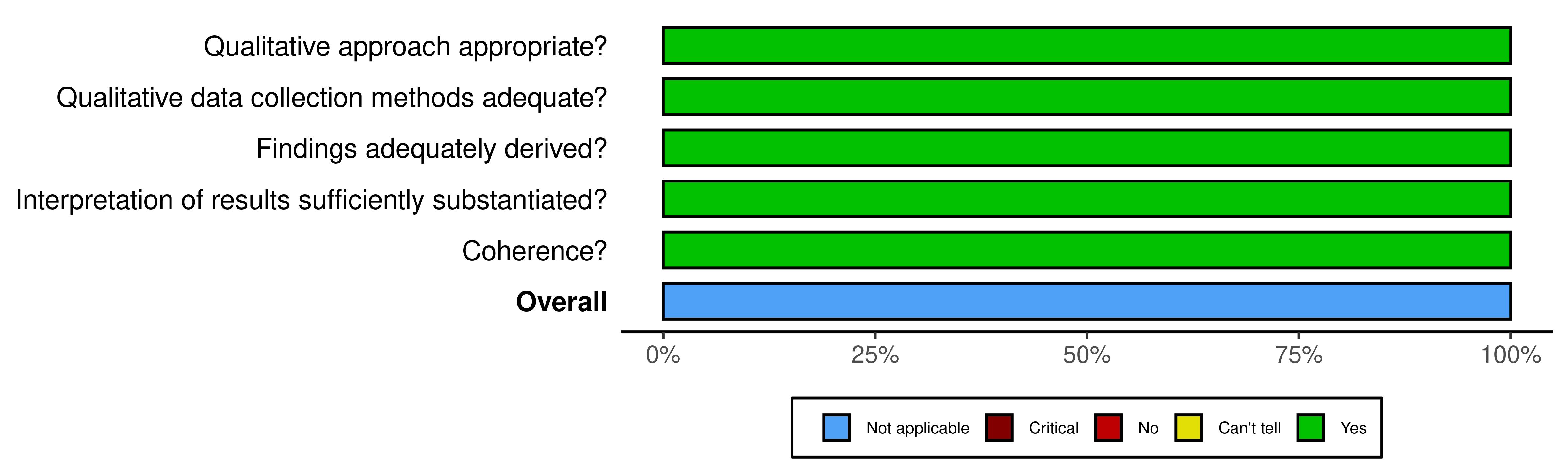


*Note.* No individual or overall scores were assigned. Adapted from McGuinness, L. A., & Higgins, J. P. T. (2020). Risk-of-bias VISualization (robvis): An R package and Shiny web app for visualizing risk-of-bias assessments. *Research Synthesis Methods*, *12*(1), 55-61. https://doi.org/10.1002/jrsm.1411

**Figure 2**

*Quantitative Descriptive Studies Summary Plot*


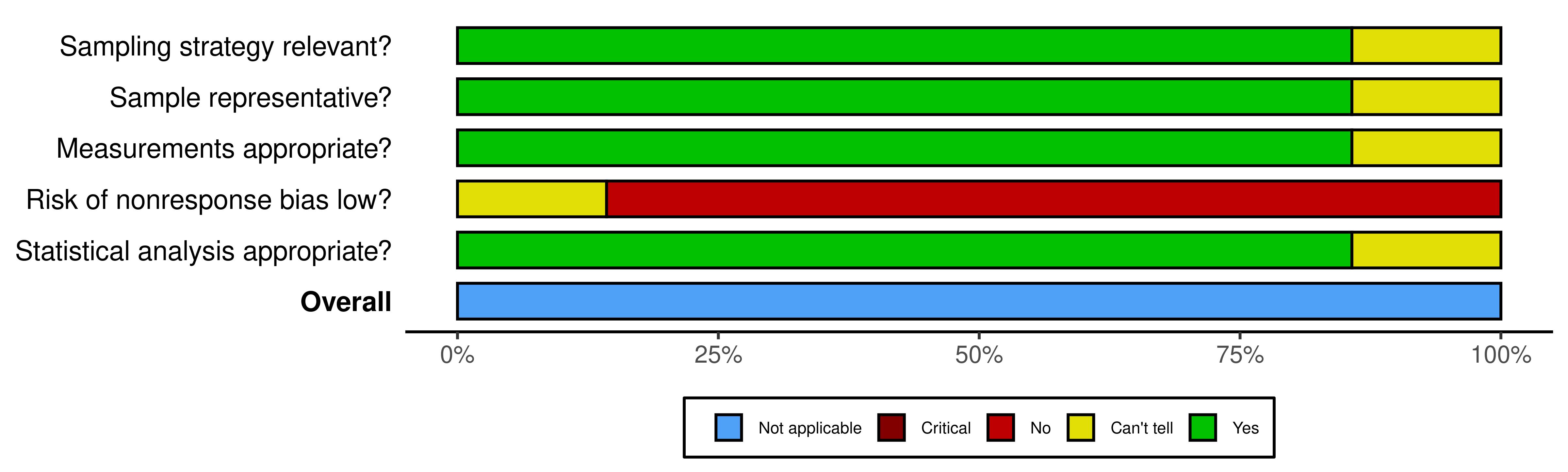


*Note.* No individual or overall scores were assigned. Adapted from McGuinness, L. A., & Higgins, J. P. T. (2020). Risk-of-bias VISualization (robvis): An R package and Shiny web app for visualizing risk-of-bias assessments. *Research Synthesis Methods*, *12*(1), 55-61. https://doi.org/10.1002/jrsm.1411

**Figure 3**

*Mixed Methods Studies Summary Plot*


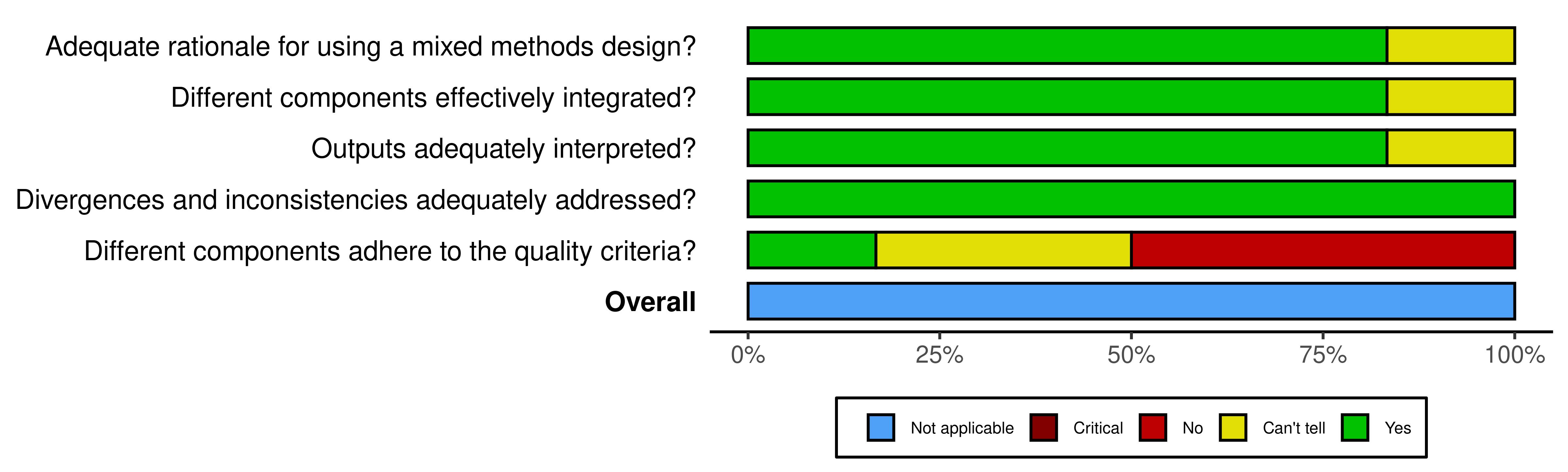


*Note.* No individual or overall scores were assigned. Adapted from McGuinness, L. A., & Higgins, J. P. T. (2020). Risk-of-bias VISualization (robvis): An R package and Shiny web app for visualizing risk-of-bias assessments. *Research Synthesis Methods*, *12*(1), 55-61. https://doi.org/10.1002/jrsm.1411

**Figure 4**

*Risk of Bias Plot*


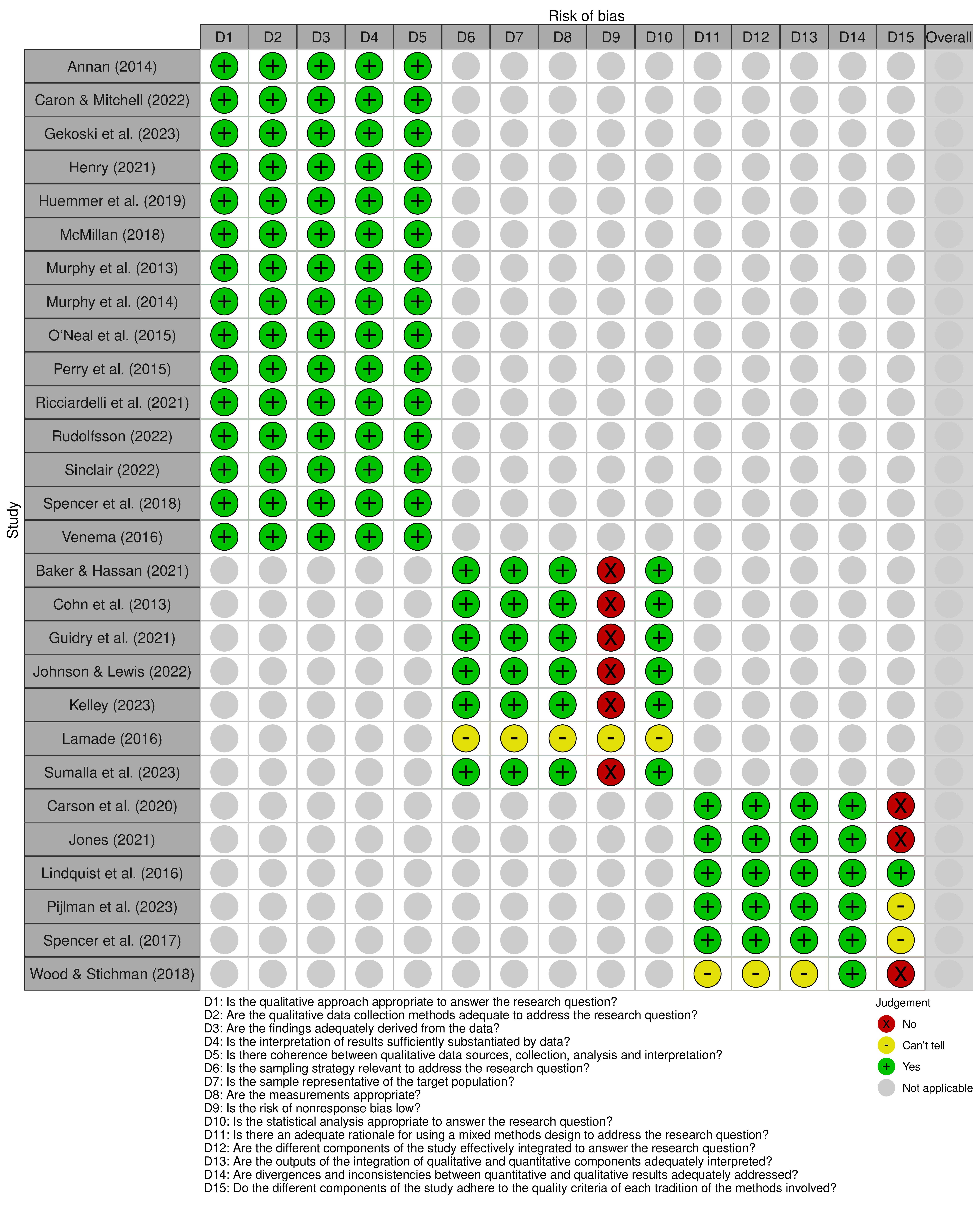


*Note.* Adapted from McGuinness, L. A., & Higgins, J. P. T. (2020). Risk-of-bias VISualization (robvis): An R package and Shiny web app for visualizing risk-of-bias assessments. *Research Synthesis Methods*, *12*(1), 55-61. https://doi.org/10.1002/jrsm.1411
